# COVID-19: Universal health coverage now more than ever

**DOI:** 10.7189/jogh.10.010350

**Published:** 2020-06

**Authors:** Benedetta Armocida, Beatrice Formenti, Francesca Palestra, Silvia Ussai, Eduardo Missoni

**Affiliations:** 1 Institute for Maternal and Child Health - IRCCS "Burlo Garofolo" - Trieste, Italy; 2 Centre for Research on Health and Social Care Management, Bocconi University, Milano, Italy

For the first time since the creation of the European Union, strategic and unprecedented measures have been taken by the Italian Government to face the COVID-19 outbreak. On 11 March 2020, the Italian Government passed the Prime Minister’s Decree, known as “Stay at home – Resto a casa” [1], which established a lockdown in the entire national territory. With the number of cases constantly growing, the Italian National Healthcare Service (Servizio Sanitario Nazionale, SSN) is proving the importance of providing Universal Health Coverage (UHC), and – at the same time – the consequences of years of definancing and privatization, fragmentation and lack of human resources.

The SSN was established in 1978 to execute the “Right to Health”, expressed by the Italian Constitution as “a fundamental right of the individual and as a collective interest, and guarantees free medical care to the indigent” [2]. It ensures universal coverage, free of charge at the point of service, for primary, secondary and tertiary care [3]. The SSN has three institutional tiers – national, regional and local – with regions and local authorities responsible for the organisation and delivery of health services, leaving the National Government with a weak strategic leadership.

The quality of the Italian universal health system, together with the healthy behaviours, contributes to favourable overall health [4]. Indeed, SSN records the second lowest preventable mortality rate in the EU, after Cyprus, and also the number of preventable deaths was one of the lowest in the EU in 2016 [5]. Despite this, since 21 February 2020, when the first case of COVID-19 was reported in Italy, the Italian SSN has faced an enormous burden. To date, 19 April 2020, with 178 972 total cases and 23 660 deaths, the health care workforce performed more than 1 million free diagnostic tests, based on a triage protocol for risk assessment.

The Lombardy region is suffering the heaviest burden of the pandemic with 66 236 COVID-19 total cases and 12 213 related deaths. Currently, the number of COVID-19 cases requiring advanced respiratory support equals 922. At its standard operational level, Lombardy’s regional system has a capacity of 724 intensive care beds [6]. However, as result of an incredible collective effort both from the public and private sectors, the system can now ensure assistance to about 1200 critical patients. A new COVID-19 dedicated Hospital, with a capacity of 400 intensive care beds, has been made available in Milan thanks to public-private partnership.

Furthermore, the local health authorities of Lombardy achieved an inter-regional agreement allowing to transfer patients needing intensive care support to other Italian regions.

To date in Italy, 80 589 individuals, who proved positive to the SARS-CoV-2 test, did not require hospitalization and are currently in self-isolation at home to avoid the jeopardization of the health care system. In addition to the network of general practitioners, also under exceptional stress, this cluster of patients is currently attended by “Special Units of Continuous Assistance”, home-care divisions each taking care of an average population of 50 000. At its standard operation level, the regular Continuous Assistance Units provide each year 16696 clinical visits per 100 000 residents.

Additionally, to avoid the national shortage of health workers, caused by decades of inadequate recruitment, the Italian Government exceptionally authorized regions to recruit a total of 5000 medical doctors, 10 000 registered nurses and 5000 social workers, allocating €660 million (US$710 million) for the purpose [7], inviting also the retired personnel and residents to support the SSN.

Although Italy provides UHC to the population largely free of charge at the point of service, over the last decade the SSN experienced financial cuts of about €37 billion (US$39 billion). The public health expenditure/GDP ratio, indicated in 6.6% for the years 2018-2020, is estimated to fall to 6.4% in 2022 [8]. Over the past ten years, decreased public funding and a prolonged period of serious economic crisis have determined a significant drop in public health care expenditure [8], as well as a gradual privatization of health care services.

During the COVID-19 pandemic, the Italian example highlights both the need of a strong political commitment and adequate emergency preparedness and the importance of a system capable of providing universal access to care.

In December 2012, the United Nations General Assembly unanimously endorsed a resolution urging countries to accelerate progress toward universal health coverage [9]. The achievement of UHC was later explicitly incorporated into the SDGs 3 targets. Two specific SDG indicators have been identified to monitor Member States progress towards UHC (i. coverage of essential services and ii. financial protection).

Health for all and an equal and adequate universal access to care has been considered a priority for international development since 1978 (Alma Ata declaration) and the importance of UHC as part of a wider Primary Health Care strategy. Indeed, the World Health Organization has made UHC the top priority for the agency and on 10 October 2019, the UN General Assembly adopted the Political declaration of the States’ commitment to achieve UHC by 2030.

Nevertheless, despite the principle of *leaving no one behind*, substantial inequalities in access to affordable quality health care remain both within and between states.

UHC is essential to build a resilient and more equitable health care system, through improving health security, increasing access to essential health services and overcoming health care inequities [10,11]. During outbreaks also, UHC protects people and vulnerable families from catastrophic financial risk, preventing impoverishment, which further contributes to infectious disease spread. Additionally, low or no financial barriers in accessing health services facilitates early case detection, identification of contacts and contributes to reduce health care expenditures related to hospitalization of severe cases [11].

In Italy, about 25% of the total population is aged 65 years and older, with 4.2% of residents living in absolute poverty and 8.2% in relative poverty (below €1085.22 [US$1150] threshold) [12]. People aged 50 and older are around 2-and-a-half times more likely to progress to a severe case of COVID-19 [13]. For severe hospitalizations, requiring intensive care support, the estimated cost per patient is about €40 000 (US$43 000) [14]. Moreover, during the current lockdown, where almost 3.7 million Italian workers [15] have lost the only source of revenue of their household, UHC protects against mortality associated with economic downturn, as unemployment is more likely associated with a higher mortality in countries without UHC [11].

**Figure:**
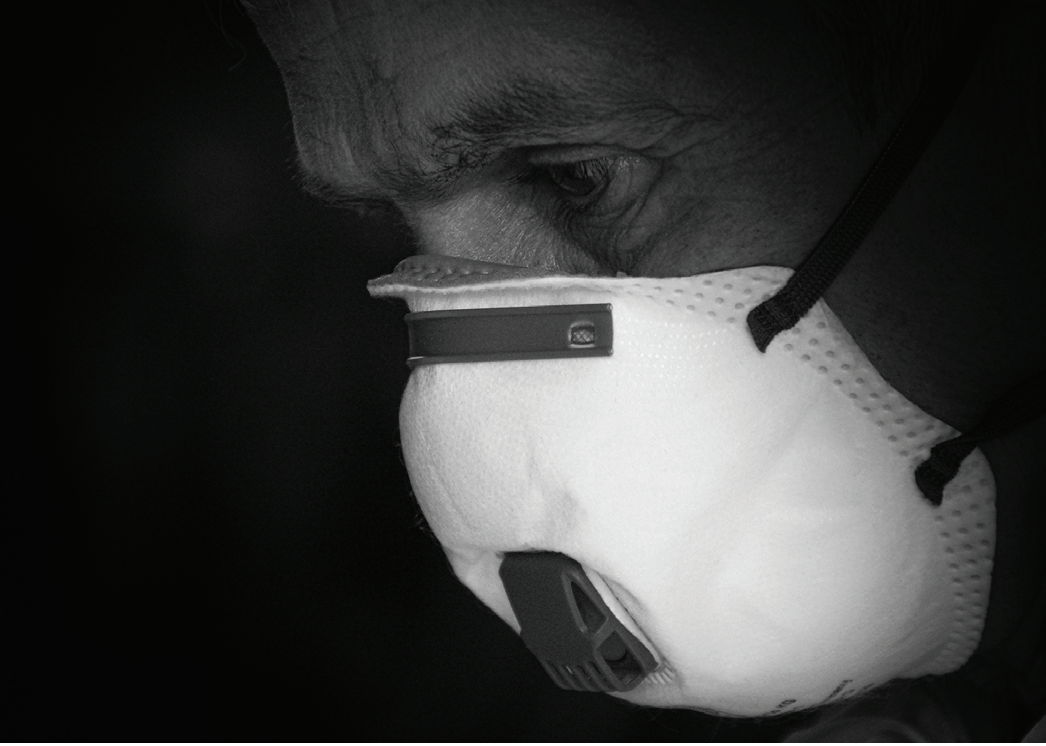
Photo: By Rottonara, available at https://pixabay.com/photos/mask-virus-pandemic-coronavirus-4934395/.

Although the UHC system in Italy guarantees free COVID-19 testing and treatment for the population and particularly the most vulnerable one, the elderly, avoiding delaying care in case of SARS-CoV-2 infection, some vulnerable groups pose particular challenges. This is the case of irregular migrants, which are estimated to be 600 000 in total [16], due to their legal status and the way they are allowed to access health services. Irregular migrants are not registered to the SSN and thus not assigned to a family doctor, still legislation provides for their access to health services through a 6-month card (STP). Due to the difficulty of accessing the health care system, including limited access to relevant information regarding their rights to care, many irregular migrants are not included in the COVID-19 response, especially in the screening programs for tracking the diffusion of infection. In addition, reduced or lack of access increases their likelihood to give up the seeking of care or to look for assistance elsewhere putting public health security at further risk [17]. A pragmatic, dignifying and inclusive response has been adopted by the Portuguese Government which recently granted permission for temporary residence to all immigrants with pending residence permit applications and asylum seekers. Granting the right to health care services, social security and financial benefits to the most fragile, as migrants, is a duty of a solidary society especially in times of crisis, such as the current epidemic [18].

In conclusion, essential quality health services must be provided to the entire population even more during exceptional events. The COVID-19 pandemic confirms the necessity of a comprehensive and inclusive UHC for individual and collective health security. Definancing, fragmenting and privatizing weaken National health systems and expose them to severe crisis in case of emergency. Governments should rather consider higher investments aimed at strengthening the community health services, epidemiological surveillance and emergency preparedness. This requires consistent management choices and a strong political commitment with a vision of a more sustainable system and resilient society.
